# Supplementary material for: Surface-reaction induced structural oscillations in the subsurface
Source: Nat Commun. 2020 Jan 16;11:305. doi: 10.1038/s41467-019-14167-1 (PMC6965640; doi:10.1038/s41467-019-14167-1)
Supplement: Supplementary file 1 — Supplementary Information [file 41467_2019_14167_MOESM1_ESM.pdf]

Supplementary Information

for

Surface-reduction induced structural oscillations in the subsurface

Xianhu Sun[1], Wenhui Zhu[1], Dongxiang Wu[1], Chaoran Li[1], Jianyu Wang[1], Yaguang Zhu[1], Xiaobo Chen[1], Jorge Anibal Boscoboinik[2], Renu Sharma[3], Guangwen Zhou[1*]

[1] Department of Mechanical Engineering & Materials Science and Engineering Program, State University of New York, Binghamton, NY 13902, USA

[2] Center for Functional Nanomaterials, Brookhaven National Laboratory, Upton, NY 11973, USA

[3] Physical Measurement Laboratory, National Institute of Standards and Technology, Gaithersburg, MD 20899, USA

[*] Correspondence to: gzhou@binghamton.edu

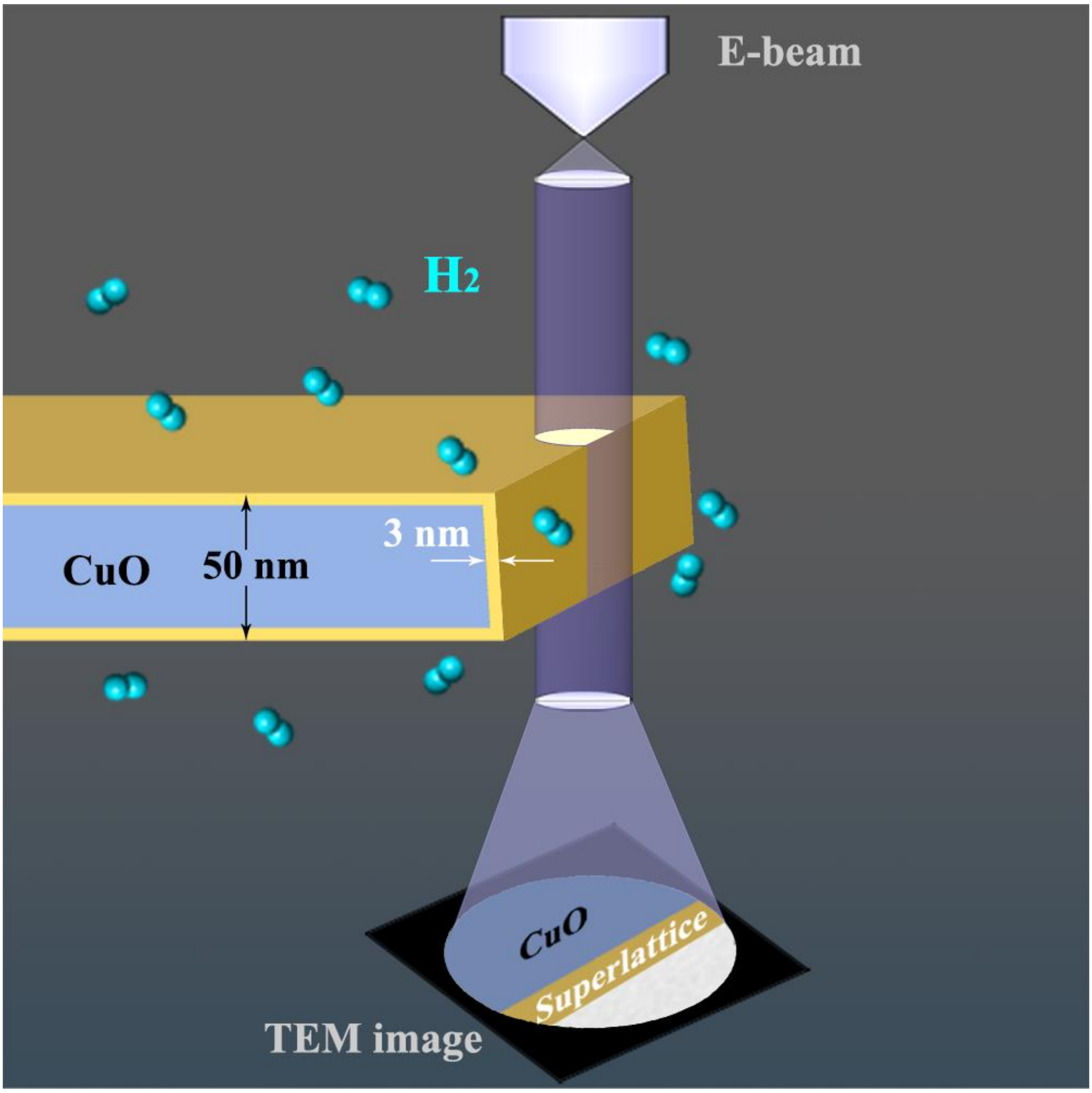

Supplementary Figure 1: Schematic illustration of in-situ TEM observations of hydrogen-adsorption induced structure evolution in CuO lattice. Dynamic structural evolution from the surface to subsurface and bulk can be visualized from the edge-on TEM imaging of the side facet of the specimen, where the specimen has a nominal thickness of ≈ 50 nm along the beam direction. Hydrogen adsorption results in structural oscillations in the subsurface region with a depth of ≈ 3 nm from the outermost surface. Noting that the planar surface is also exposed to hydrogen gas, but any structure evolution at the planar surface and in the subsurface region cannot be readily resolved from plan-view TEM imaging, where the image contrast is bulk-dominated.

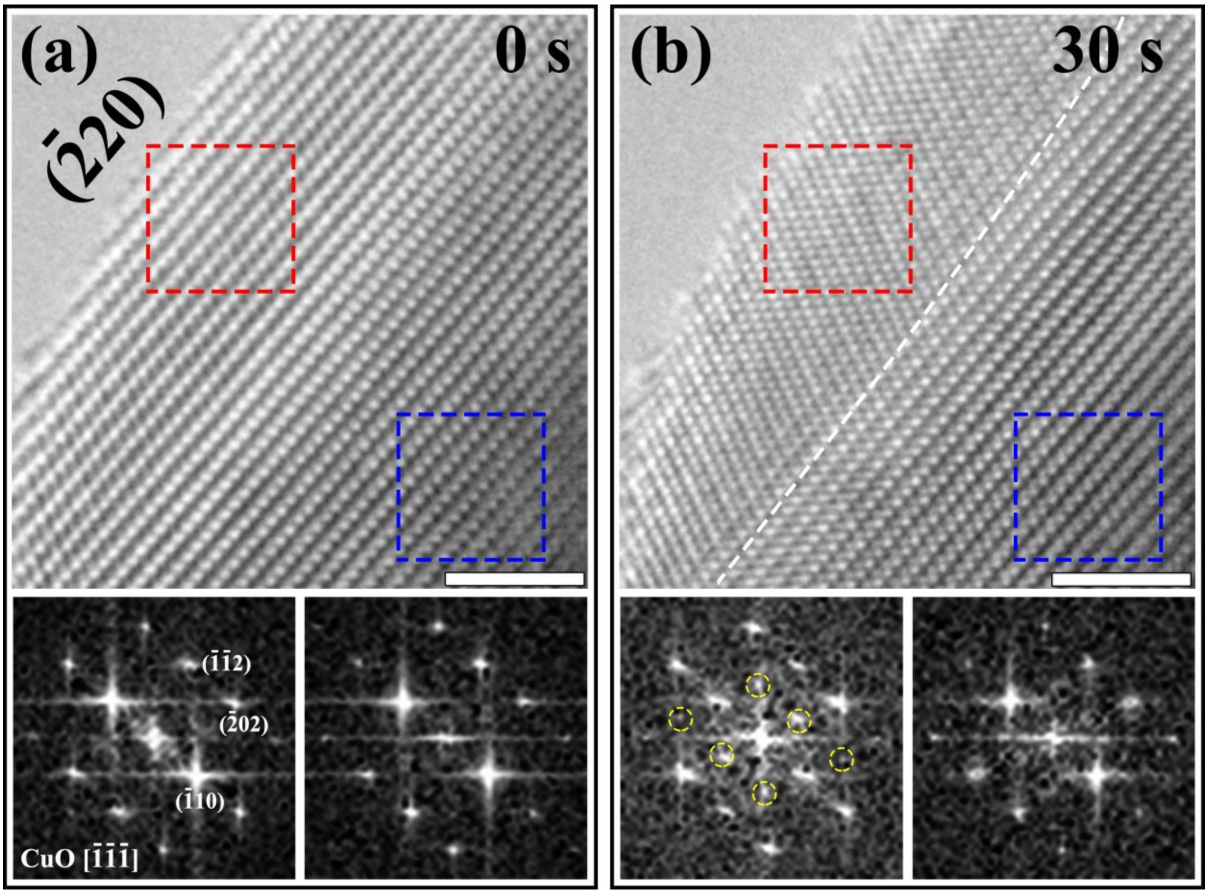

Supplementary Figure 2: Atomic-scale observation of the CuO lattice under vacuum annealing and in H_2 gas flow. (a) Upper panel: HRTEM image of the CuO lattice during vacuum annealing at 300 °C. Lower panel: Diffractograms obtained from the subsurface and deeper regions, as marked by the red and blue dashed boxes, showing the absence of superlattice spots in both regions. (b) Upper panel: HRTEM image of the CuO lattice after 30 s of H_2 gas flow at T $\approx$ 300 °C and pH_2 $\approx$ 0.5 Pa, where the dashed line marks roughly the boundary between the superlattice region and the deeper unaffected region. Lower panel: Diffractograms obtained from the subsurface and deeper regions, as marked by the red and blue dashed boxes, showing the subsurface transforms to the superlattice feature while the deeper region stays unaffected. *Scale bar*, 2 nm

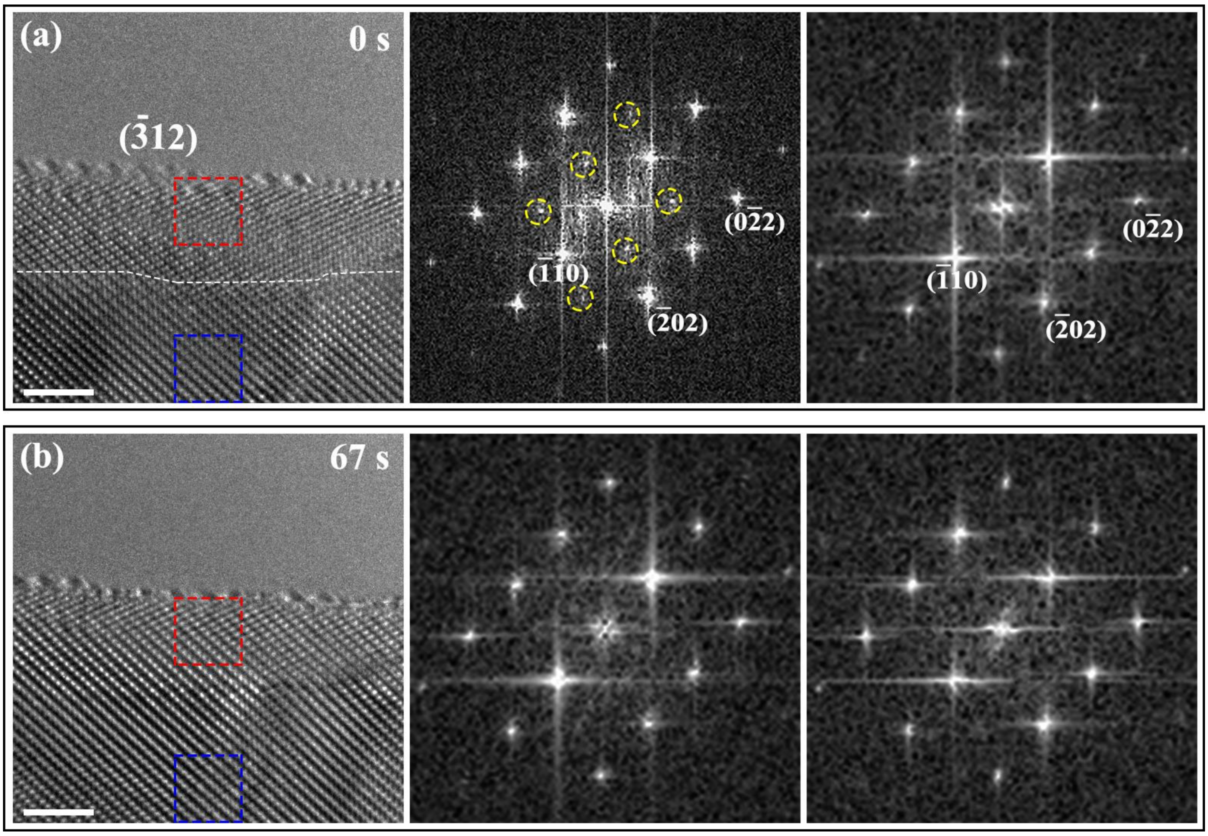

Supplementary Figure 3: Excluding the electron beam irradiation effect on the observed structural oscillations during the H_2 exposure at T ≈ 300 °C and pH_2 ≈ 0.5 Pa. (a) Left: HRTEM image showing a $(\bar{3}12)$ surface with the presence of the superlattice feature in the subsurface region. The dashed line marks roughly the boundary between the superlattice region and the deeper unaffected region. Middle: Diffractogram obtained from the subsurface marked by the red dashed box, where superlattice spots (marked by dashed yellow circles) are visible. Right: Diffractogram obtained from the deeper, bulk region, as marked by the blue dashed box, in which only the fundamental spots are present. (b) Left: the e-beam was blanked for ~ 67 s and then unblanked for TEM imaging, showing that the superlattice contrast in the subsurface region has disappeared without the electron irradiation. Middle: Diffractograms from the subsurface region, as marked by the red dashed box, in which the superlattice spots are barely visible. Right: Diffractogram from the deeper region as marked by the dashed blue box, in which only the fundamental spots are visible. *Scale bar*, 2 nm

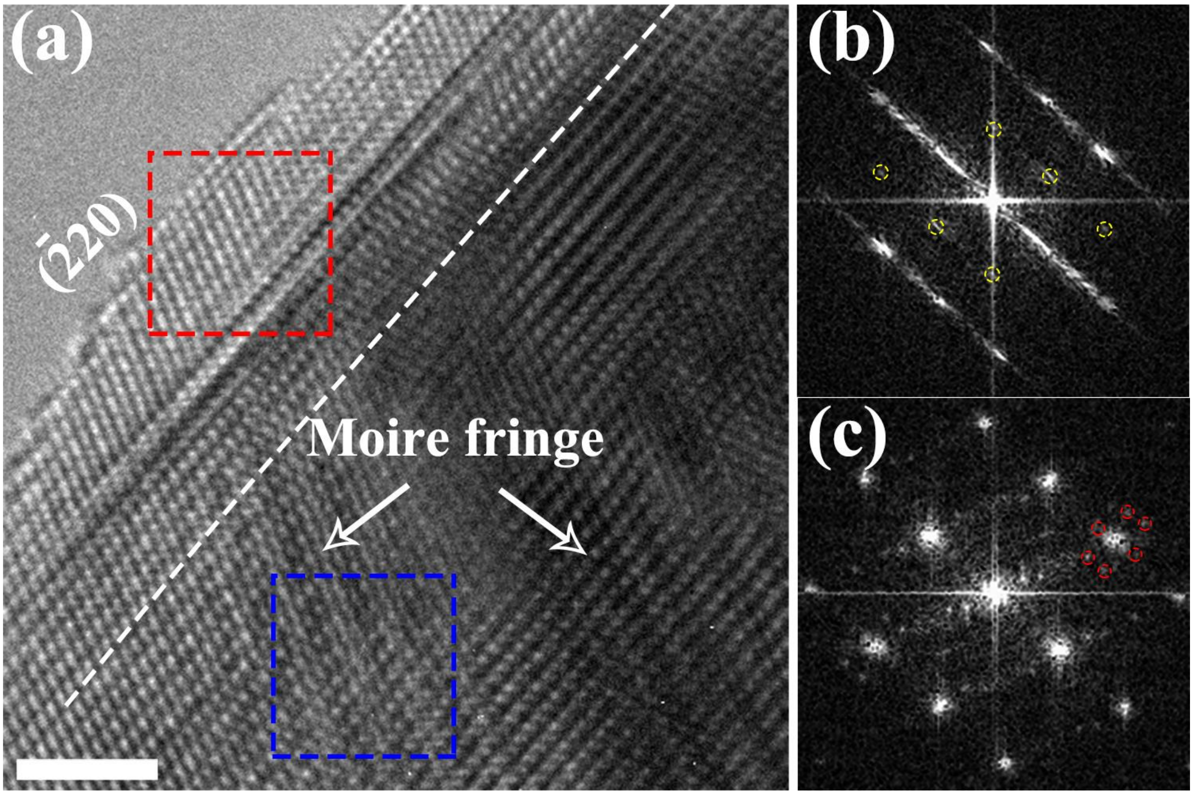

Supplementary Figure 4: Aggregation of Cu atoms released from the H_2 adsorption induced decay of surface steps during H_2 exposure at T ≈ 300 °C and pH_2 ≈ 0.5 Pa. (a) HRTEM image showing the presence of the moiré fringe contrast as a result of the formation of a Cu overlayer on the inner surface region of the specimen via the aggregation of Cu adatoms released from the decay of atomic steps at the CuO surface. The dashed line marks roughly the boundary between the superlattice region and the deeper unaffected region. (b) Diffractograms from the subsurface region as marked by the dashed red box, in which superlattice spots from the ordering of oxygen vacancies are visible, as marked by the dashed yellow circles. (c) Diffractogram from the deeper (bulk) region, in which the superlattice spots are absent but double diffraction spots due to the overlapped lattices of Cu and CuO are visible, as marked by the small red circles. *Scale bar*, 2 nm

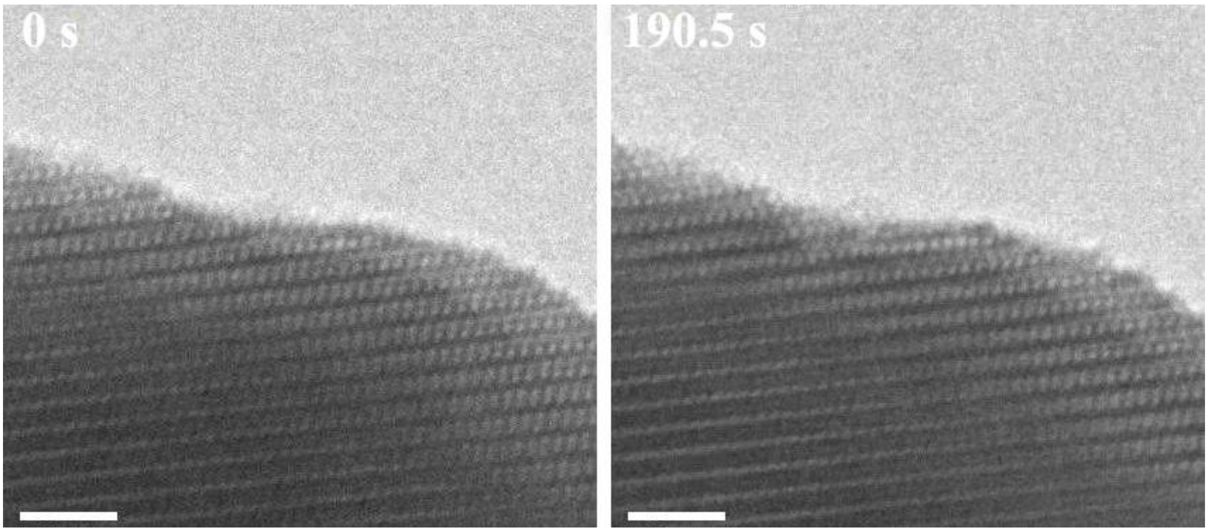

Supplementary Figure 5: Time-sequence HRTEM images (Supplementary Movie 4) showing no structural oscillations within a time period of 190.5 s for a CuO sample at 300 °C and 0.01 Pa of H_2 gas flow. This suggests that the observable structural oscillations require a reasonably fast generation rate of oxygen vacancies in the CuO lattice. The slow surface reaction kinetics (due to the low H_2 pressure) may result in significant dilution of the concentration of oxygen vacancies across a large depth of the sample, which does not induce observable structure changes. *Scale bar,* 2 nm

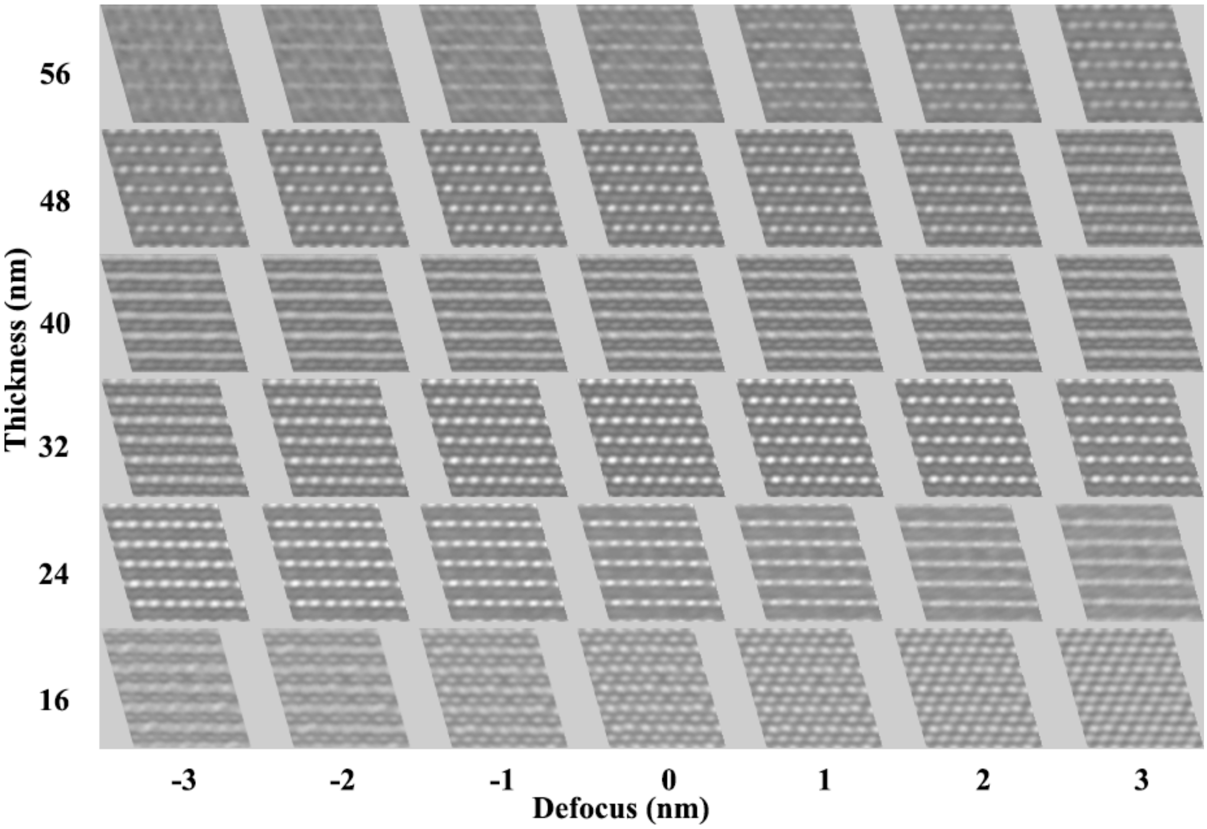

Supplementary Figure 6: Simulated HRTEM images of perfect CuO viewed along the $[\bar{1}\bar{1}\bar{1}]$ direction. Specimen thickness increases from bottom to top and defocus increases from left to right.

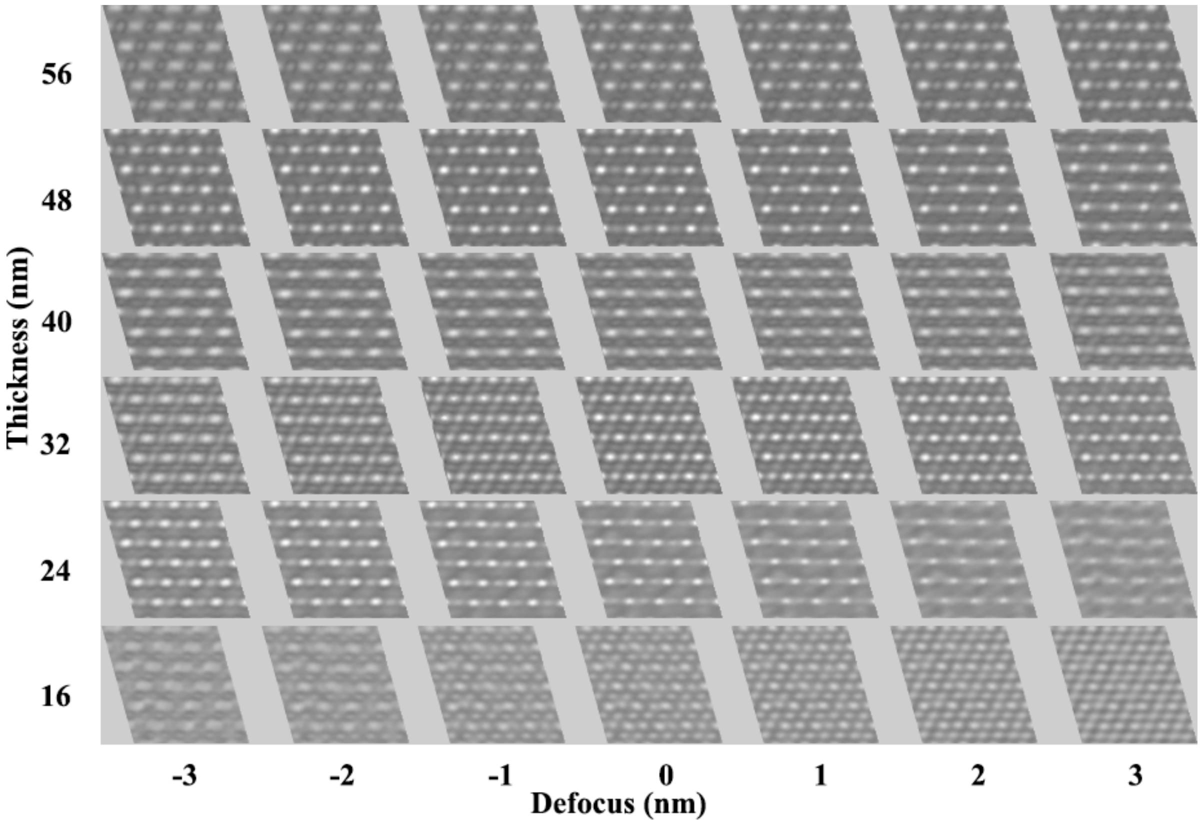

Supplementary Figure 7: Simulated HRTEM images of the vacancy-containing CuO viewed along the $[\overline{111}]$ direction. Specimen thickness increases from bottom to top and defocus increases from left to right.

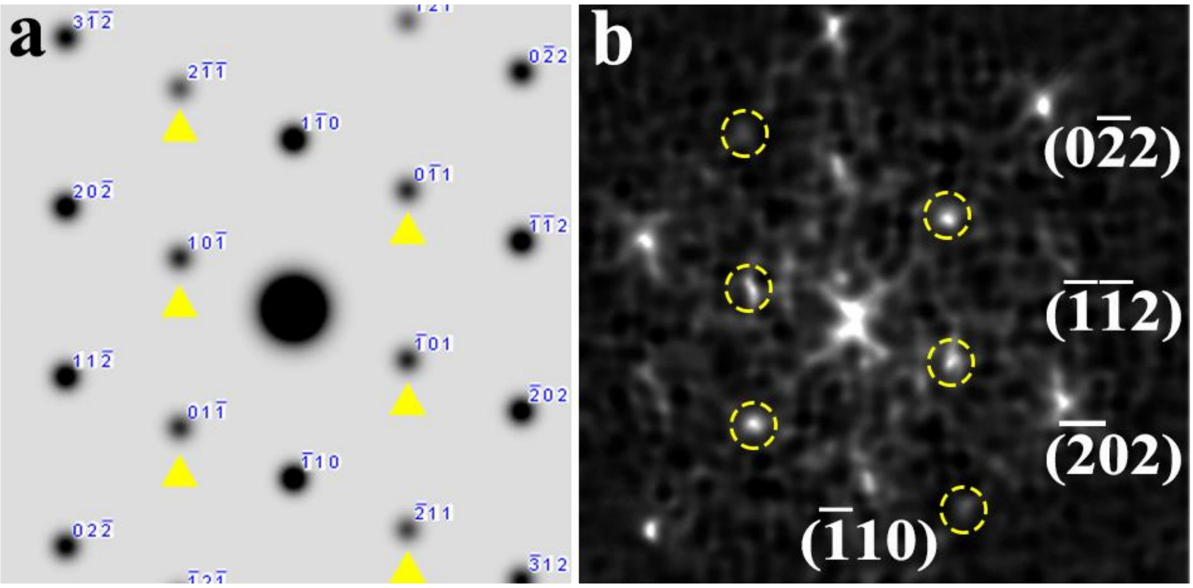

Supplementary Figure 8: Comparison between simulated and experimental diffractograms. (a) Simulated $[\overline{1}11]$ zone axis electron diffraction pattern of the DFT-obtained superstructure in Figure 4(e**).** The superlattice diffraction spots are labeled by yellow triangles, the other diffraction spots correspond to the parent phase of CuO. **(b)** Experimental diffractogram in Figure 1(f). Superlattice spots are marked with dashed yellow circles.
