# Supplementary material for: Surface-reaction induced structural oscillations in the subsurface
Source: Nat Commun. 2020 Jan 16;11:305. doi: 10.1038/s41467-019-14167-1 (PMC6965640; doi:10.1038/s41467-019-14167-1)
Supplement: Supplementary file 3 — Description of Additional Supplementary Files [file 41467_2019_14167_MOESM3_ESM.pdf]

Description of Additional Supplementary Files

File Name: Supplementary Movie 1
Description: In situ TEM video showing cyclic formation of superlattice contrast in CuO during the continuous H_2 flow at T = 300 °C and $pH_2 \approx 0.5$ Pa, where the surface display a high density of steps and terraces. The video is accelerated by a factor of 3 of the real time.

File Name: Supplementary Movie 2
Description: In situ TEM video showing structural oscillations in the subsurface of CuO during the continuous H_2 flow at T = 300 °C and $pH_2 \approx 0.5$ Pa, where the surface consists of atomic steps. The video is accelerated by a factor of 3 of the real time.

File Name: Supplementary Movie 3
Description: In situ TEM video showing the cyclic formation of superlattice contrast in CuO during the continuous H_2 flow at T = 300 °C and $pH_2 \approx 0.5$ Pa, where the majority of the surface is atomically flat. The video is accelerated by a factor of 3 of the real time.

File Name: Supplementary Movie 4
Description: In situ TEM video showing the absence of structural oscillations in CuO at T = 300 °C and $pH_2 \approx 0.01$ Pa. The video is accelerated by a factor of 5 of the real time.
